# Prostate volume and biopsy tumor length are significant predictors for classical and redefined insignificant cancer on prostatectomy specimens in Japanese men with favorable pathologic features on biopsy

**DOI:** 10.1186/1471-2490-14-43

**Published:** 2014-05-29

**Authors:** Masahiro Yashi, Tomoya Mizuno, Hideo Yuki, Akinori Masuda, Tsunehito Kambara, Hironori Betsunoh, Hideyuki Abe, Yoshitatsu Fukabori, Osamu Muraishi, Koyu Suzuki, Yoshimasa Nakazato, Takao Kamai

**Affiliations:** 1 Department of Urology, Dokkyo Medical University, 880 Kitakobayashi, Mibu, Shimotsuga, Tochigi 321-0293, Japan; 2 Department of Urology, St. Luke’s International Hospital, Tokyo, Japan; 3 Department of Pathology, St. Luke’s International Hospital, Tokyo, Japan; 4 Department of Pathology, Dokkyo Medical University, Tochigi, Japan

**Keywords:** Predictive factor, Insignificant cancer, Index tumor volume, Prostate volume, Biopsy tumor length

## Abstract

**Background:**

Gleason pattern 3 less often has molecular abnormalities and often behaves indolent. It is controversial whether low grade small foci of prostate cancer (PCa) on biopsy could avoid immediate treatment or not, because substantial cases harbor unfavorable pathologic results on prostatectomy specimens. This study was designed to identify clinical predictors for classical and redefined insignificant cancer on prostatectomy specimens in Japanese men with favorable pathologic features on biopsy.

**Methods:**

Retrospective review of 1040 PCa Japanese patients underwent radical prostatectomy between 2006 and 2013. Of those, 170 patients (16.3%) met the inclusion criteria of clinical stage ≤ cT2a, Gleason score (GS) ≤ 6, up to two positive biopsies, and no more than 50% of cancer involvement in any core. The associations between preoperative data and unfavorable pathologic results of prostatectomy specimens, and oncological outcome were analyzed. The definition of insignificant cancer consisted of pathologic stage ≤ pT2, GS ≤ 6, and an index tumor volume < 0.5 mL (classical) or 1.3 mL (redefined).

**Results:**

Pathologic stage ≥ pT3, upgraded GS, index tumor volume ≥ 0.5 mL, and ≥ 1.3 mL were detected in 25 (14.7%), 77 (45.3%), 83 (48.8%), and 53 patients (31.2%), respectively. Less than half of cases had classical (41.2%) and redefined (47.6%) insignificant cancer. The 5-year recurrence-free survival was 86.8%, and the insignificant cancers essentially did not relapse regardless of the surgical margin status. MRI-estimated prostate volume, tumor length on biopsy, prostate-specific antigen density (PSAD), and findings of magnetic resonance imaging were associated with the presence of classical and redefined insignificant cancer. Large prostate volume and short tumor length on biopsy remained as independent predictors in multivariate analysis.

**Conclusions:**

Favorable features of biopsy often are followed by adverse pathologic findings on prostatectomy specimens despite fulfilling the established criteria. The finding that prostate volume is important does not simply mirror many other studies showing PSAD is important, and the clinical criteria for risk assessment before definitive therapy or active surveillance should incorporate these significant factors other than clinical T-staging or PSAD to minimize under-estimation of cancer in Japanese patients with low-risk PCa.

## Background

The widespread use of prostate-specific antigen (PSA) screening and multiple core biopsy protocol resulted in early detection of prostate cancer (PCa) at a curable stage, and was associated with dramatic decrease in PCa mortality in North America and Europe [1]. The European Randomized Study of Screening for Prostate Cancer (ERSPC) trial showed that PSA-based screening significantly reduced mortality by 21% [2]. The analysis of cancer trends using the national cancer mortality data in Japan also revealed the similar stage migration of PCa. The incidence of localized cancer increased markedly between 2000 and 2003 with an annual percent change of 29.7%, then became stable, while PCa mortality began to decrease in 2004 [3]. The early detection of PCa consequently raises the new issue that the proportion of low-risk cancers for which the definitive therapy will not alter prognosis has been increasing. Potential side effects after definitive therapies for localized cancer worsen the patients’ quality of life [4], even with the current advantages of robot-assisted surgery or image-guided radiation therapy.

Gleason pattern 3 less often has molecular abnormalities, so called cancer hallmarks and often behaves indolent compared to Gleason pattern 4 in PCa [5,6]. Although all patients underwent prostatectomy and it was uncertain how therapeutic an effect it had, the 15-year cancer specific mortality rate for pathologic Gleason score (GS) 6 or less was reported as 0.2% [7]. On the other hand, it is controversial whether the low grade and low volume PCa within a few positive biopsy cores could avoid immediate definitive therapy or not, because some cases harbor unfavorable pathologic features at radical prostatectomy specimens with a variety of rates [8]. To predict clinically insignificant cancer in patients with clinical T1c (non-palpable) PCa, Epstein et al. reported a set of criteria [9], and later updated to the contemporary version, including PSA density < 0.15 ng/mL, biopsy Gleason score ≤ 6, the presence of tumor in two or fewer cores, and no more than 50% involvement by tumor in any core [10]. The review of validation studies on Epstein criteria concluded that it had suboptimal accuracy for predicting insignificant cancer from significant heterogeneity in the results of insignificant cancer, GS ≤ 6, and organ-confined status at 37–76%, 54.3–75.9% and 80.0–96.9%, respectively [11]. Currently, active surveillance (AS) has become one of the key treatment options as a strategy for deferring treatment for low-risk PCa in American and European urological associations’ guidelines, but the presence of several AS protocols consisting of different clinic-pathologic factors complicate decision making of physicians and patients.

In view of the racial differences, the clinical criteria developed from Western cohort analysis could not be directly applied to Japanese or Asian patients [12]. In addition, the updated definition of index tumor volume threshold to 1.3 mL and total tumor volume threshold to 2.5 mL for insignificant cancer raises reconsideration of the current risk assessment before therapy [13]. To our knowledge, the study using this updated definition has still been insufficient. In this study, we investigated the associations between preoperative clinical data and pathologic results of prostatectomy specimens along with oncological outcome to identify predictors for classical and redefined insignificant cancer in Japanese men who met our expanded inclusion criteria.

## Methods

### Inclusion criteria of patients

The study population consisted of 1040 consecutive patients that underwent radical prostatectomy between January 2006 and December 2013 at 2 Japanese academic institutions. We retrospectively reviewed the records for those pathologic findings of multiple core biopsy and clinical stages. Of those, 170 patients met our inclusion criteria of clinical stage ≤ cT2a [14], GS ≤ 6 without Gleason pattern 4 or 5 as secondary scores, up to two biopsies with cancer, and no more than 50% of cancer involvement in any core; no limitation was set on PSA value and PSA density (PSAD). None of the patients had received hormonal treatments including antiandrogens, luteinizing hormone-releasing hormone analogues, or 5-alpha reductase inhibitors preoperatively.

### Preoperative clinical data including biopsy and radiographic image

Preoperative patient characteristics are provided in Table 1; All prostate volume was measured by magnetic resonance imaging (MRI), which was more accurate than transrectal ultrasound and computed tomography for volume estimation [15], and PSAD was determined as pre-biopsy PSA value divided by MRI-estimated prostate volume. The mean number of biopsy cores per procedure was 13.1 and 19 cases (11.2%) had fewer than 10 cores. Biopsy specimens obtained by transrectal and transperineal approaches were evaluated for GS, number of cores involved with cancer, total length of tissue, and length of cancer measured with subtracting the intervening benign glands. Gleason scoring of the biopsy specimens was done according to the International Society of Urological Pathology (ISUP) Consensus 2005; the second most prevalent pattern to the highest cancer grade observable in the specimen [16]. MRI findings were simply classified according to report by radiologists whether there were typical suspicious lesions for malignancy or not. Seventy patients (41.2%) fulfilled the contemporary Epstein criteria [10], and 103 patients (60.6%) fulfilled the criteria of the Prostate Cancer Research International: Active Surveillance (PRIAS) study [17]. The PRIAS criteria includes clinical stage ≤ T2, PSA ≤10 ng/mL, PSAD <0.2 ng/ml/cc, ≤2 positive cores, and GS ≤ 6.

**Table 1:** Preoperative patient characteristic

	**Number (%) or Mean value (range)**	
Number	170 (100)	
Age (year)		65.5 (40 to 78)
PSA (ng/ml)		7.4 (2.8 to 25.9)
Prostate volume (cc)		40.2 (13.8 to 87.9)
PSA density (ng/ml/cc)		0.208 (0.050 to 1.124)
Biospy core (n)		13.1 (6 to 24)
Tumor length (mm)		2.1 (0.1 to 7.0)
Biopsy approach		
Transrectal	137 (80.6)	
Transperineal	33 (19.4)	
Positive core		
1	120 (70.6)	
2	50 (29.4)	
Biopsy Gleason score		
5	7 (4.1)	
6	163 (95.9)	
Clinical T stage(DRE)		
cT1c	154 (90.6)	
cT2a	16 (9.4)	
MRI findings		
Negative	113 (66.5)	
Positive	57 (33.5)	
Epstein criteria		
Yes	70 (41.2)	
No	100 (58.8)	
PRIAS criteria		
Yes	103 (60.6)	
No	67 (39.4)	

*Abbreviations: DRE* dicital rectal examination, *PRIAS* Prostate Cancer Research International: Active Surveillance.

### Evaluation of prostatectomy specimens

Prostatectomy specimens obtained through 127 open retropubic surgery (74.7%) and 43 through robot-assisted surgery (25.3%) were processed according to the Stanford protocol [18], step sectioned transversely at 5 mm intervals, and mounted as half or quarter sections for microscopic evaluation. Those were evaluated for GS, extraprostatic extension, surgical margin status, seminal vesicle invasion, lymph node involvement when dissection was performed, and tumor volume. Gleason scoring was also done as recommended in the ISUP Consensus 2005 [16]. Prostate cancer volume was calculated from the three-dimensional measurements of the dominant nodule (index tumor) that correlates with oncological outcome better than total tumor volume [19], using a spherical formula and correcting by shrinkage factor (1.33) due to formalin fixation. Two specialists of urologic pathology from the 2 institutions reported the histopathology of biopsy and prostatectomy specimens, and we retrospectively reviewed the reports.

### Definitions

To avoid confusion of terminology, we describe the following definitions. Classical insignificant cancer was defined by a pathologic stage ≤ pT2, GS ≤ 6, and an index tumor volume < 0.5 mL [8]. Redefined insignificant cancer was characterized as a pathologic stage ≤ pT2, GS ≤ 6, and an updated index tumor volume threshold of 1.3 mL [13]. Both insignificant cancers are results confirmed on prostatectomy specimens. In addition, Epstein criteria and PRIAS criteria are established sets of preoperative factors to predict insignificant cancer [10,17]. Biochemical recurrence was defined as PSA level greater than 0.2 ng/mL with subsequent PSA rising.

### Analyses and statistics

The associations between preoperative clinical data and pathologic characteristics of prostatectomy specimens for targeting the unfavorable pathologic results, and oncological outcome were analyzed. The quantitative data were categorized into two groups by median values, and the qualitative data were compared using a chi-squared test or Fisher’s exact test. Recurrence-free survival was estimated using the Kaplan–Meier method and differences were compared with the log-rank test. Logistic regression and Cox proportional hazards regression model were used for multivariate analyses. All statistical analyses were performed with EZR, which is a graphical user interface for R (The R Foundation for Statistical Computing, version 2.13.0). All statistical tests were two-sided, with *p*-value of less than 0.05 considered to be statistically significant.

### Ethics statement

This study was conducted in accordance with the Helsinki Declaration and was approved by the institutional ethical review boards at Dokkyo Medical University Hospital and St. Luke’s International Hospital. In addition, each patient signed a consent form with regard to the storage of their information for the purpose of research.

## Results

Tumor characteristics of prostatectomy specimens are provided in Table 2. Pathologic stage ≥ pT3, positive surgical margin (PSM), upgraded GS, index tumor volume ≥ 0.5 mL, and ≥ 1.3 mL were detected in 25 patients (14.7%), 29 patients (17.1%), 77 patients (45.3%), 83 patients (48.8%), and 53 patients (31.2%), respectively. Less than half of cases had classical (41.2%) and redefined (47.6%) insignificant cancer. Of those with stage ≥ pT3, 23 patients had extraprostatic extension, and 4 patients had seminal vesicle invasion. No lymph node involvement was observed among 158 patients (92.9%) who underwent lymph node dissection that was limited in the obturator areas. In 7 patients, pT0 cancers, namely “vanishing cancer” in prostatectomy specimens were observed. These cases were accounted as insignificant cancers for the following analysis.

**Table 2:** Tumor Characteristic of prostatectomy specimens

	**Number (%) or Mean value (range)**	
Number	170 (100)	
Pathologic T stage		
pT0	7 (4.1)	
pT2	138 (80.2)	
pT3a	20 (11.8)	
pT3b	4 (2.4)	
pT4	1 (0.6)	
Surgical margin		
Negative	141 (82.9)	
Positive	29 (17.1)	
Prostatectomy Gleason score		
≤6	86 (50.6)	
7	71 (41.8)	
8	6 (3.5)	
NA	7 (4.1)	
Index tumor volume (cc)		1.42 (0 to 14.62)
<0.5	87 (51.2)	
0.5-1.3	30 (17.6)	
>1.3	53 (31.2)	
Classical insignificant cancer		
Yes	70 (41.2)	
No	100 (58.8)	
Redefined insignificant cancer		
Yes	81 (47.6)	
No	89 (52.4)	

*Abbreviations: NA* not available due to pT0.

**Figure 1:**
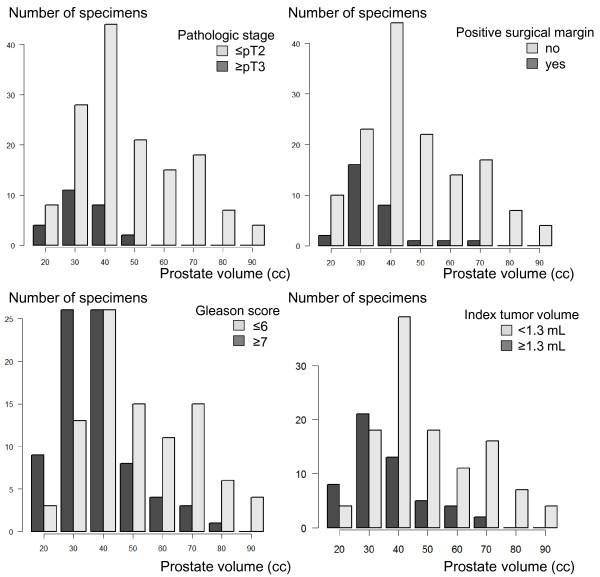
**Prostate volume distribution in relation to unfavorable pathologic results on prostatectomy specimens.** The patients with prostate volume larger than 43.3 cc never presented with pathologic stage ≥ pT3, and rarely presented with PSM. Prostate volume ≥35.5 cc predicted stage ≤ pT2 (p = 0.0004) and negative surgical margins (p = 0.0009), namely organ-confined cancers.

**Figure 2:**
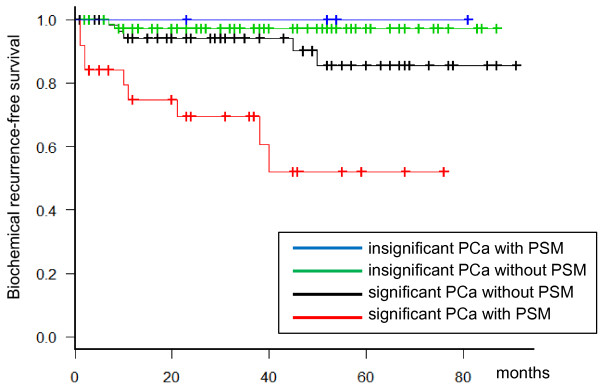
**Kaplan–Meier event curves presenting biochemical recurrence-free survivals for clinically significant/insignificant cancer with or without positive surgical margin.** The patients with redefined insignificant cancers (pathologic stage ≤ pT2, GS ≤ 6, and index tumor volume < 1.3 mL) essentially did not relapse regardless of the surgical margin status (only 2 cases without PSM relapsed).

Univariate analyses of preoperative factors revealed that the MRI-estimated prostate volume, PSAD, biopsy tumor length, MRI findings, contemporary Epstein criteria, and PRIAS criteria were all or partly associated with unfavorable pathologic results on prostatectomy specimens (Table 3), and all factors were associated with the presence of insignificant cancer of both definitions (Table 4). Meanwhile patient age, PSA value, number of biopsy cores or positive cores, biopsy Gleason score 5 or 6, difference in biopsy approach or institution, and clinical T-stage determined by digital rectal examination did not hold any associations with unfavorable pathologic results. Multivariate model excluding the established preoperative criteria such as Epstein and PRIAS criteria showed that both large prostate volume (≥35.5 cc) and short tumor length on biopsy (≤2.0 mm) showed independent predictive value for both classical and redefined insignificant cancer. PSAD showed independent value for only redefined insignificant cancer (Table 4). Figure 1 shows the profiles of prostate volume distribution in relation to the unfavorable pathologic features, and the patients with prostate volume larger than 43.3 cc never presented with pathologic stage ≥ pT3, and rarely presented with PSM (only 2 cases).During a median follow-up of 39.5 months (interquartile range 17.3-58.0), 16 patients developed biochemical recurrence. The estimated 5-year recurrence-free survival and cancer-specific survival were 87.0% and 100%, respectively. PSM was the only independent factor among unfavorable pathologic results. Figure 2 shows the Kaplan–Meier event curves for biochemical recurrence-free survivals. The patients with redefined insignificant cancers essentially did not relapse regardless of the surgical margin status, but 2 cases (2.5%) without PSM relapsed and consequently received salvage radiotherapy.

**Table 3:** Preoperative factors associated with unfavorable pathologic results on prostatectomy specimens

**Factors**	**pT-stage**			**Surgical margin**			**Gleason score**			**Index tumor volume**					
	**≤pT2**	**≥pT3**	***p*-value**	**neg**	**posi**	***p*-value**	**≤6**	**≥7**	***p*-value**	**<0.5 mL**	**≥0.5 mL**	***p*-value**	**<1.3 mL**	**≥1.3 mL**	***p*-value**
Prostate volume (cc)															
≥35.5	81	4	0.0004	79	6	0.0009	61	24	<0.0001	60	25	<0.0001	71	14	<0.0001
<35.5	64	21		62	23		32	53		27	58		46	39	
PSA density (ng/ml/cc)															
<0.172	78	7	0.0228	78	7	0.0037	58	27	0.0007	56	29	0.0002	69	16	0.0008
≥0.172	67	18		63	22		35	50		31	54		48	37	
Tumor length (mm)															
≤2.0	107	11	0.0045	102	16	0.0788	76	42	0.0002	74	44	<0.0001	88	30	0.0194
>2.0	38	14		39	13		17	35		13	39		29	23	
MRI findings															
Positive	99	14	0.2560	99	14	0.0305	69	44	0.0227	68	45	0.0011	83	30	0.0801
Negative	46	11		42	15		24	33		19	38		34	23	
Epstein criteria															
Yes	63	7	0.1880	65	5	0.0038	48	22	0.0029	49	21	<0.0001	57	13	0.0040
No	82	18		76	24		45	55		38	63		60	40	
PRIAS criteria															
Yes	91	21	0.1870	90	13	0.0633	65	38	0.0075	64	39	0.0005	79	24	0.0070
No	54	13		51	16		28	39		23	44		38	39	

*Abbreviations: neg* negative, *posi* positive.

**Table 4:** Univariate and multivariate analyses of factors predicting classical and redefined insignificant cancer

	**Classical insignificant cancer**						**Redefined insignificant cancer**					
**Factors**	**Univariate model**			**Multivariate model**			**Univariate model**			**Multivariate model**		
	**OR**	**95% CI**	***p*-value**	**OR**	**95% CI**	***p*-value**	**OR**	**95% CI**	***p*-value**	**OR**	**95% CI**	***p*-value**
Prostate volume (≥35.5 vs.<35.5)	5.87	2.97-11.60	<0.0001	4.59	2.13-9.90	0.0001	5.79	2.99-11.20	<0.0001	4.31	2.07-8.98	<0.0001
PSA density (<0.172 vs. ≥0.172)	3.71	1.94-7.10	<0.0001	2.12	0.99-4.53	0.0533	3.74	1.98-7.06	<0.0001	2.11	1.01-4.40	0.0460
Tumor length (≤2.0 vs. >2.0)	5.11	2.29-11.40	<0.0001	5.41	2.21-13.2	0.0001	3.57	1.75-7.27	0.0005	3.46	1.56-7.69	0.0023
MRI findings (neg vs. posi)	2.43	1.22-4.83	0.0148	2.13	0.95-4.75	0.0663	2.17	1.12-4.19	0.0210	1.84	0.86-3.94	0.1150
Epstein criteria (yes vs. no)	3.46	1.82-6.58	0.0002				3.20	1.69-6.05	0.0003			
PRIAS criteria (yes vs. no)	2.48	1.28-4.78	0.0068				2.47	1.30-4.66	0.0055			

*Abbreviations: OR* Odds ratio, *95% CI* 95% confidence interval, *neg* negative, *posi* positive.

When statistics were limited in 70 patients who met the contemporary Epstein criteria, the rate of stage ≥ pT3, PSM, upgraded GS, index tumor volume ≥ 1.3 mL, and redefined insignificant cancer reduced to 10.0%, 7.1%, 31.4%, 18.6%, and 64.3%, respectively. In 103 patients who met criteria of the PRIAS study, those rates were 11.7%, 12.6%, 36.9%, 23.3%, and 56.3%, respectively. Furthermore, in 32 patients that were classified as intermediate risk or more because of a PSA value > 10 ng/mL, the rate of redefined insignificant cancer was 43.8%.

## Discussion

Our study demonstrated that prostate volume and biopsy tumor length had independent value for predicting both classical and redefined insignificant cancer, and PSAD showed the independent value for only the redefined insignificant cancer. The different statistics between classical and redefined insignificant cancers in the multivariate analysis might imply that PSAD possibly holds predictive power in larger or aggressive tumors. Substantial overlaps existed in cases with small prostate volume, but large prostate volume firmly had high positive predictive value for tumors of stage ≤ pT2 and negative surgical margins, namely organ-confined cancers. We consider that the finding that prostate volume is important does not simply mirror many other studies showing that PSAD is important. Furthermore, clinical T-staging had little value, and the multiparametric MRI would possibly add some diagnostic value if evaluation was performed in detail.

The favorable features on multiple core biopsies consistently harbor unfavorable pathologic results on prostatectomy specimens despite fulfilling the established criteria developed in North America and Europe. Certainly, in our data, patients meeting Epstein and PRIAS criteria harbored clinically significant cancer for one-third and around half of cases, respectively. Oon et al. speculated that the modification to Gleason scoring might be associated with a reduced accuracy of Epstein criteria, because distinct differences were observed in validation studies between pre- and post-2005 [11,18]. In addition, Wolters, et al. presented a recent analysis using a data set from a randomized screening trial that demonstrated that clinically insignificant prostate cancer may include GS 6, pT2 tumors with index tumor volumes of up to 1.3 mL [13] instead of 0.5 mL, which had been used as a threshold for around 20 years [20]. These critical alterations suggest reconsideration of the current methods used for risk assessment before definitive therapy or AS. The established criteria consisting of clinical T-staging by digital rectal examination or PSAD would not be satisfactory for patients in this study, and the clinical criteria should be compiled incorporating the prostate size and biopsy length involved by cancer.

Prostate volume has been less often mentioned than PSAD or PSA as a predictive factor despite that all of these parameters held independent values [21]. PSAD is a comprehensive parameter considering both serum PSA and prostate size, but the value varies easily based on the fluctuation of PSA. In that sense, prostate volume estimated using MRI is a stable preoperative parameter and the only tumor-unrelated factor in our analysis model. Despite the variations of study design and whether the prostate volume was estimated pre- or postoperatively, small prostate volume is unanimously reported with association between the poor oncological outcomes in several studies. Freedland et al. reported that more high-grade and more advanced cancers were detected in men with smaller prostates along with lower serum testosterone concentrations in a large population ranging from clinical T1 to T3 cancers and suggested that prostate size might be an important prognostic variable that should be evaluated for use pre- and postoperatively [22]. Tilki et al. and Chung et al. reported the associations especially in relation to GS upgrading [21,23]. These trends were also observed even when study populations were limited with GS ≤ 6 by Milonas et al. [24] and with highly selective criteria (T1c, PSA < 10 ng/mL, a single positive biopsy, tumor length < 3 mm, and Gleason score < 7) by Beauval et al. [25]. The prostate volume would be directly affected by age and endocrine factors [26], and the mean prostate size should be significantly different between Japanese and Western populations even after adjusting for differences in age, height, and weight [27]. Although the median prostate volume was obviously different between 35.5 cc in our study and more than 40 to 50 cc in the Western study, our study confirmed that prostate volume retained its predictive ability for both classical and redefined insignificant cancer in Japanese men.

The percentage and length of cancer involvement in biopsy core are also significant predictors, and have already been incorporated in the major prediction models before developing AS protocols [28]. Russo et al. reported that inclusion of the percentage of cancer involvement contributed to reducing the misclassification in patients eligible for AS according to the PRIAS criteria, which does not reference any cancer involvement in the core [29]. Antonelli et al. used the updated definition of total tumor volume and determined its optimal cutoff to be 20% for the diagnosis of insignificant cancer using the receiver operating characteristic curve [30]. Freedland et al. reported the same threshold of 20% for predicting PSA recurrence after prostatectomy [31]. We agree with their strict threshold despite the fact that our data were analyzed in terms of tumor length but not in the percentage of tumor involvement, and we consider the threshold of 50%, which is used in the many AS protocols, to be too relaxed to avoid under-estimation of cancer. However, a more stringent threshold, namely minute or microfocal cancer defined by ≤ 5% or ≤ 1 mm in a single core, is not a guarantee of insignificant cancer [8].

The current established clinical criteria can-not eliminate the risk of over- and under- estimation of cancer. A stringent selection criteria excludes a considerable number of patients who are willing to be managed by AS, even those having potentially insignificant cancer, and therefore, well-balanced criteria between sensitivity and specificity are required for patients. The implication of increasing the index tumor volume threshold to 1.3 mL is that we should miss small-volume cancer and set the AS protocols to be more expanded. In the recent report of head-to-head comparison of contemporary AS protocols, Iremashvili et al. revealed that the PRIAS and University of Miami criteria demonstrated the best balance between sensitivity and specificity for insignificant prostate cancer among the existing AS protocols, and the contemporary Epstein criteria demonstrated high specificity but low sensitivity for all end points [32].

Nevertheless, clinically significant cancer might not always progress, and some cases might remain indolent for a substantial duration of time. A validation of AS protocol should not be a surrogate endpoint, such as the analysis of the pathologic results of prostatectomy specimens, but should be a long-term outcome of a prospective cohort. According to the review of AS in the large prospective series, approximately one-third of patients were treated after a median surveillance of about 2.5 years because of histologic reclassification on biopsy or a PSA doubling time of less than 3 years, while some cases were treated with no evidence of progression [33]. The short- to mid-term estimated treatment-free survivals were reported as 62 to 72% at 5 years and at 43 to 62% at 10 years [34,35]. These data characterize AS as a strategy for deferring treatment and justify it as an optimal choice for patients with low-risk PCa that can accept the confirmatory biopsy within 1 to 2 years and the slight increased risk of late metastasis.

To develop a model to discriminate clinically indolent from aggressive disease efficiently, advances in biochemical markers replacing PSA or PSA derivatives such as prostate cancer antigen 3 or transmembrane protease serine 2 will be required in addition to the existing factors [36]. In addition, a more detailed analysis of multiparametric MRI, including number of lesions, lesion suspicion, and lesion density (calculated as total lesion volume/prostate volume) [37], and image-guided targeted biopsy should play a positive role [38]. The current study has some limitations; there is no control population outside of Japan other than the published literature. It is a retrospective study based on a relatively small Japanese population, and the pathologic examination was performed at 2 institutions. The median follow-up time was also relatively short to determine oncological outcomes. Thus, the results may not apply to the Western population. Nonetheless, it is valuable to give insight into ethnic differences, and these data provide useful information that could help predict insignificant cancer in Japanese or Asian patients with favorable features on needle biopsy. The findings of this study should be validated in a larger, independent dataset.

## Conclusions

The favorable features of biopsy often are followed by adverse pathologic findings on prostatectomy specimens despite fulfilling the established criteria. Large prostate volume and short tumor length on biopsy remained as independent predictors for classical and redefined insignificant prostate cancer in Japanese patients with favorable pathologic features on needle biopsy. The clinical criteria for risk assessment before definitive therapy or AS should incorporate these factors to minimize under-estimation of cancer in Japanese patients with low-risk PCa.

## Competing interests

The authors declare that they have no competing interests.

## Authors’ contributions

MY, TM and OM initiated this study, participated in its design and coordination, carried out the study, performed the statistical analysis. MY, TM drafted the manuscript. HY, AM, TK, HB, HA, YF, KS, YN and TK carried out the study. All authors read and approved the final manuscript.

## Pre-publication history

The pre-publication history for this paper can be accessed here:

http://www.biomedcentral.com/1471-2490/14/43/prepub
